# “Looking back to my family”: Indigenous Australian patients’ experience of hemodialysis

**DOI:** 10.1186/1471-2369-13-114

**Published:** 2012-09-20

**Authors:** Kate Anderson, Joan Cunningham, Jeannie Devitt, Cilla Preece, Alan Cass

**Affiliations:** 1 The George Institute for Global Health, Sydney, NSW, Australia; 2 Menzies School of Health Research, Charles Darwin University, Darwin, NT, Australia; 3 Sydney Medical School, University of Sydney, Sydney, NSW, Australia; 4 Independent consultant anthropologist, Darwin, NT, Australia

**Keywords:** Indigenous Australian, Hemodialysis, End stage kidney disease, Access to healthcare, Life experiences, Patient care, Health communication, Late diagnosis, Qualitative research

## Abstract

**Background:**

In common with Indigenous populations elsewhere, Indigenous Australians have higher incidence of end-stage kidney disease (ESKD), but lower transplantation rates than their non-Indigenous counterparts. Understanding how the demands of dialysis impact on, and are impacted by, the lives of Indigenous patients may provide important insight into treatment pathways and decision-making.

**Methods:**

We conducted semi-structured interviews in 2005–06 with 146 Indigenous and 95 non-Indigenous patients from nine hospital renal wards and 17 associated dialysis centres, which together treat the majority of Indigenous Australian ESKD patients.

**Results:**

Factors influencing treatment experience included: the impacts of late diagnosis; family separations associated with relocating for treatment; the physical and psychosocial demands of hemodialysis; and ineffective communication between health care providers and patients. Although not unique to them, Indigenous patients were more likely to experience the combined effect of *all* factors.

**Conclusions:**

Social/situational circumstances profoundly affect Indigenous Australian dialysis patients’ ability to fully engage with treatment. This may ultimately affect their likelihood of receiving optimal treatment, including transplantation. Areas for improvement include: earlier diagnosis; improved linkages between specialist renal services and primary care in regional settings; more effective communication and patient education; and more systematic, transparent approaches to patient “compliance” in transplant and home dialysis guidelines.

## Background

In common with Indigenous populations in the US, Canada and New Zealand, Indigenous Australians have higher incidence of end-stage kidney disease (ESKD), but lower transplantation rates than their non-Indigenous counterparts [1-4]. For example, in 2005, 45.4% of non-Indigenous treated ESKD patients in Australia had a functioning transplant, compared with only 12.7% of treated Indigenous patients; corresponding figures for 2009 were 45.9% and 12.0% [1]. Reasons for these disparities are not completely understood, but clinical factors alone do not explain them.

Most Indigenous Australian ESKD patients attend in-centre hemodialysis [1], generally in large urban centres. Many patients from regional/remote areas must leave home – often suddenly and indefinitely – to access treatment. The hemodialysis regimen is extremely demanding, with on-going dialysis attendance, tight dietary restrictions and multiple medications. Much of a dialysis patient’s life is spent travelling to/from dialysis sessions, having treatment, and recovering afterwards.

Time and resource constraints in clinical settings mean that healthcare providers make decisions about patients often with limited understanding of their individual circumstances [5]. For ESKD patients, the impact of such decisions can be profound and potentially life-changing, determining, for example, whether a patient is assessed as suitable for home-based dialysis or transplantation. Many Indigenous ESKD patients share social and situational circumstances likely to affect their responses to the demands of dialysis, including remote/regional residence, low income and education, and a first language other than English [6-8]. These factors – *taken together* – significantly increase the challenges of dialysis [9,10]. This may explain, at least in part, characterisations of some Indigenous patients as “non-compliant” with treatment, a label with negative impacts on patients’ access to the full range of renal replacement modalities [11,12].

Given the vulnerability of this patient group, it is important to understand how dialysis treatments impact on, and, in turn, are impacted by the lives of Indigenous patients. This paper presents the views and experiences of Indigenous Australian ESKD patients undergoing dialysis, comparing them with those of non-Indigenous patients. It draws on material from a large in-depth interview study involving patients from around Australia.

## Methods

Data presented here were collected as part of IMPAKT (Improving Access to Kidney Transplants), an integrated, mixed-methods program of work investigating barriers faced by Indigenous ESKD patients in accessing kidney transplants. Brief methodological details relating to the present analysis are described below. A more detailed account of IMPAKT’s aims, methodology, ethical issues, recruitment, sampling and data analysis is available elsewhere [13].

### Data collection

Semi-structured interviews were conducted in 2005–06 with 146 Indigenous and 95 non-Indigenous patients from nine hospital renal wards and 17 associated dialysis centres, which together treat the majority of Indigenous Australian ESKD patients. All participants provided informed consent. A maximum diversity sampling strategy helped select patients based on ethnicity, location, age, sex, treatment type, and illness duration. The interview structure aimed to elicit a life-story narrative that made sense to the patient [14]. Topics included personal history of illness, social and psychosocial context, attitudes to treatments including transplantation, adequacy of information and communication, and satisfaction with services. Almost all interviews were conducted individually and face-to-face by one of three investigators (JD, CP, KA) and digitally recorded and transcribed. Most interviews were conducted in English. In order to elicit more nuanced perceptions and attitudes from some patients for whom English was not their first language, seven interviews were conducted entirely in an Indigenous language by fluent non-Indigenous contract interviewers.

### Analysis

Thematic analysis was performed using QSR NVivo 7 (QSR International, Melbourne, Australia). The analytical methods used were evolutionary and iterative in nature [15,16]. The research team met regularly throughout the study to propose, debate and negotiate the major thematic groups arising from the interview material. One investigator (KA) coded all the interviews for themes relating to patients’ experience of dialysis and engagement with treatment. Participant demographics were self-reported. Descriptive statistics were generated using SPSS 15.0 for Windows (SPSS, Chicago, Illinois).

### Ethical approval

The study was approved by 14 relevant jurisdictional ethics committees, including: Aboriginal Health & Medical Research Council; Aboriginal Health Research Ethics Committee of the Aboriginal Health Council of South Australia; Cairns Base Hospital Ethics Committee; Central Australian Human Research Ethics Committee; Central Sydney Area Health Service Ethics Review Committee; Department of Human Services (South Australia) Ethics Committee; Human Research Ethic Committee of the Northern Territory Department of Health & Community Services and the Menzies School of Health Research; Macquarie and Far West Area Health Service Human Research Ethics Committee; North Western Adelaide Health Service Human Research Ethics Committee; Princess Alexandra Hospital Ethics Committee; Royal Perth Hospital Ethic Committee; Townsville Health Service District Institutional Ethics Committee; Western Australian Aboriginal Health and Information Ethics Committee; and Wuchopperen Health Services Ethics Committee. Site-based reference groups represented staff and institutional interests.

## Results

### Participant characteristics

Compared with non-Indigenous participants, Indigenous participants were more likely to be female, younger and have dependents, and less likely to speak English as their first language, be employed, or have completed secondary school (Table 1). The majority of Indigenous patients (71%) lived in a remote area (generally without dialysis facilities) at the time of diagnosis, compared with 15% of non-Indigenous participants.

**Table 1:** Characteristics of indigenous and non-indigenous participants (n = 241)

	**Indigenous (n = 146) %**	**Non-indigenous (n = 95) %**
Female	524	42
Age group (years)		
20-39	16	15
40-59	64	52
60+	19	34
Married or in a de facto relationship	56	56
Has dependents	52	32
Completed secondary school or higher	14	38
Currently employed*	10	39
First language is English	42	86
Reads English not very well or not at all	27	7
Has a car	32	90
Has a phone	62	98
Uses the internet	9	46
Remote/very remote residence prior to diagnosis	71	15
Treatment modality		
Centre- or satellite-based hemodialysis	87	63
Home hemodialysis or peritoneal dialysis	10	18
Functioning transplant	3	19

* Includes full-time, part-time or casual employment.

### Patients’ experiences of dialysis

Patient interviews suggested several factors shaped their treatment experience including: the impacts of late diagnosis; the consequences of family separations necessitated by moving to treatment centres; the physical and psychosocial demands of dialysis; and, ineffective communication between patients and their care providers. None of these factors were unique to Indigenous patients: some non-Indigenous patients also had communication difficulties, some were also diagnosed at a late, acute stage, some endured family separations and so on. But, in stark contrast to Indigenous patients none were likely to experience *the combined, interactive and continuing effects of all these factors*. The conjunction of treatment-related circumstances, with a profile of disadvantage and social marginalisation, posed a substantial challenge for Indigenous patients to engage with their treatments.

### Late, unplanned start on dialysis

Many Indigenous participants described an abrupt, unplanned commencement of dialysis. This was often accompanied by immediate relocation to a distant town/city, with no time to mentally or physically prepare; in many cases, patients were unaware of the severity of their kidney problems. The following example hints at the profound impact of late diagnosis:

"Interviewer (I): Okay, so you were there with your kids and just got sick one day - and then what happened?"

"Participant (P): That’s when they fly me down to [major city]…[I was there for] three and a half years – yes, might be more. (Indigenous male patient, age 70+ years)"

Recalling their first experiences of dialysis, Indigenous patients described shock, fear and bewilderment:

"P: I was shocked first. Yeah, shocked because nobody had ever said anything to me about [kidney disease] … I went to the hospital and when I got there [it was] late - late at night in the hospital. And the next day the doctor came and had a look at me and they were looking at my - you know - which side they’re gonna put [the fistula]. I didn’t say anything, I was wondering what they was doing, you know, I thought somebody might come up and tell me about it. Then when it was time for them to do it, that’s when the doctor said that they was gonna put a tube down here [pointing to site of vascath access in neck]. And they didn’t say anything [to explain] that they was getting me ready [for dialysis]… I had to find out myself."

"They put that thing on, and then I was wondering, ”What that’s for”, you know? …they put me in a wheelchair to this part [of the hospital] where they had dialysis. I looked and I see this thing one side of me and then I said - I was thinking to myself: “So this is what it’s like to be on the machine”. (Indigenous female patient, age 60–69 years)"

By contrast, most non-Indigenous patients were aware of and being monitored for loss of kidney function well before commencing dialysis. This tended to lessen the shock of starting treatment.

"P: I mean I was expecting it you know, for some time, and so [I was] very prepared, yeah. Well, if it’s an acute thing, I can understand it’s probably a huge shock to people, and things like that, but … I was quite prepared, even though when they said, “Oh look you’ve got to go on dialysis next week”, it was sort of that I knew it was going to happen, but it was something I would sort of rather not heard. I just didn’t want it to interrupt my lifestyle and things like that mainly. (Anglo-Australian female patient, age 50–59 years)"

### Family separation through relocating for treatment

Most Indigenous participants had had to leave home permanently to access dialysis treatment. Thrice weekly dialysis sessions severely limit opportunities to visit distant home communities. Indigenous participants spoke of the sometimes overwhelming difficulties of constant separation from their families, communities and land.

"P: I am living without my children, living far away from them. I have had to leave my home behind, and the lands, and am living in [major town] away from my friends and family. I have been living [here, having dialysis] a long time. So I would like [the doctors] to think about what I have said, to discuss the situation and put us on the [transplant] list. If [an Indigenous patient] gets another kidney and everything goes okay on return, he or she would be able to go home to family - see the children. To go home - this is what a lot of [Indigenous people] want. They don’t want to be waiting for such a long time, they are getting really weary, the poor things. (Indigenous male patient, age 60–69 years, translated from Indigenous language)"

Indigenous patients were, on the whole, younger than non-Indigenous patients as well as more likely to be caring for dependent family members, including children. Although some non-Indigenous participants were also caring for children, they were less likely to have relocated for treatment. Indigenous parents faced a grim choice between bringing their children with them to an unfamiliar town/city life or leaving them behind in the care of others. Finding housing, social networks and support services for families and children all present difficulties; many patients expressed concerns about exposing their families to alcohol and drugs and/or the likelihood of homelessness. The alternative was a life undergoing a difficult treatment in virtual exile from their families, communities and support networks.

In addition to family responsibilities, dialysis requirements may cut across or conflict with patients’ family, community and/or cultural responsibilities. Patients described the dilemma of having their personal health needs set against their social and cultural responsibilities.

"P: I’m a very special person [there] with the other young men because they take part in special ceremonies. It’s very important for my people. And I felt - about the ceremony - I felt guilty in myself because I’m not over there. I got a lot of phone calls - the Land Council, the [community] Council - my people ring me up and say, “Oh, we need you to come over for this funeral”. And I keep saying to them, “I can't travel”. That’s the word I’m getting to them …I feel no good when I keep saying, “No” to my people. My wife and I, we both talked over many times… She said “Listen, you can't keep going there for ceremony, otherwise you [will] have a heart attack or stroke or something… Tell your people you got to think about your health first. It’s very important.” (Indigenous male patient, age 40–49 years)"

This patient was a senior community member with ceremonial obligations to younger kin. He asks: how can I put my own health above my responsibilities to my family and kin? His far-distant relatives have no comparable experience to really understand his situation – he feels guilty and uncaring. On the other hand, travelling and thus missing treatments flies in the face of his health carers’ advice and the pleas of his wife.

### Physical and psychosocial demands of treatment

All participants spoke of a range of physical and/or psychosocial demands associated with the dialysis treatment itself, including fear, pain, nausea, fatigue, faintness, and a range of negative feelings such as deep sadness, loneliness, and homesickness.

"P: Because of the time lying there on the [dialysis] machines, four hours like that … the back becomes really painful… When it’s finished, the sisters pull out the needles and get everything, give us medicine. And the patients feel paralysed - they can’t go[walk]. They feel really tired. The bus driver has to lift them up into the bus. When they get off the bus they move slowly using walking frames. … So it’s because of all this that people are sad. Also the dialysis affects their eyesight - after the machine some can’t see properly. They recover later after going home and having a sleep… The dialysis machine has some bad side effects. (Indigenous male patient, age 60–69 years, translated from Indigenous language)"

While non-Indigenous patients also noted some physical discomforts associated with dialysis, their commentary did not reflect a similar experience of distress, with its suggestions of alienation, social isolation, confusion, and sadness.

### Ineffective communication

Communication difficulties, associated variously with differences of language, literacy, conceptual frameworks, values, and preferred communication styles between patients and health providers, were recurrent themes of Indigenous commentary. Patients noted that medical jargon, and/or overly complex English, made it difficult for them to understand information and instructions. This was particularly so for specialists. Some participants reported apprehension and confusion as a result. Others spoke of feeling uncomfortable and uncertain in the hospital environment, which reduced their willingness to engage with health professionals. As an experienced Indigenous interpreter observed:

"People [i.e. Indigenous patients] will go along and shake their head and agree to things [just] to get away from that person [clinician] that’s firing all these big questions at them …[but] they’re lost for answers. They can’t understand all that. So the easiest way is to agree … because you know you’re out [quickly] then. … And [the health providers are] … happy; … Oh well at least they feel as if they’ve done their job so they [feel] good about it. But the full story hasn’t been given."

Perceiving clinicians as reluctant to provide information, several Indigenous participants expressed uncertainty and/or scepticism about whether health staff wanted to give them the whole story. This was keenly felt by some:

"P: … When I ask the doctor I don't get anything [clear information]. Have I got something else wrong [with me]? The doctors keep it a secret, they hide it. We want them to tell us plainly, “This is the problem”. They don't talk. (Indigenous female patient, age 50–59 years, translated from Indigenous language)"

Moreover, the busyness of the daily dialysis routine can itself mask a lack of patient understanding and participation in their own care. Asked what he knew of his current situation, a longer term Indigenous patient replied:

"P: Well, we don’t know. We really only just go in and out and have the treatments. We don’t know where we stand with how we’re going. So we don’t know whether we’re getting a bit better or things are getting a bit worse. They don’t tell us whether we’re improving or getting worse. We’re just going in and out of the sessions. (Indigenous male patient, age 40–49 years, translated from Indigenous language)"

In contrast, only a small proportion of non-Indigenous patients did not speak English as their first language. Most reported few difficulties understanding their health providers; those who did report difficulties focused primarily on language differences with little mention of medical jargon or overly complex language. Notably, while most major treating units routinely used interpreter services for non-Indigenous, non-English languages, only one unit involved in this study made regular use of Indigenous language interpreter services.

### Maintaining the dialysis regimen

Despite the challenges most Indigenous patients described themselves as following their health providers’ advice with its emphasis on attending dialysis and maintaining dietary and medication requirements. Those who reported being unable or unwilling to maintain the regimen gave a variety of reasons, including transport problems, not understanding what was expected, difficulty in adjusting to dialysis, needing to travel home for important events, and feelings of mistrust, anger or frustration.

Although Indigenous patients commonly noted that information from their care providers had been inadequate, either in its comprehensiveness or, more usually, its appropriateness (e.g. diet-related advice), most also expressed a reticence about asking for clarification or additional information.

Several Indigenous patients recalled how anger and frustration at their vastly changed circumstances led to neglecting their treatment:

"I: Okay, so you have missed your dialysis on some days? Only in the initial part when you were getting used to coming here?"

"P: Coming here, yeah, because I was really agitated, angry. (Indigenous male patient, age 40–49 years)"

Others explained how they tested treatment boundaries:

"P: I mucked up … for a little while there, I didn’t come to dialysis."

"I: What made you change your mind about it?"

"P: Well, because of the body, how the body functions. While I’m not being dialysed it’s becoming poison inside my body. Toxins. And that’s what I realised… I went five days without treatment. It was stupid I think but I wanted to push the limit to see how far I can go too… (Indigenous male patient, age 40–49 years)"

After the initial shock of starting dialysis and adjusting to its physical demands, many Indigenous participants described coming later to the *full realisation* that their need for treatment was *permanent* – that despite prolonged and aggressive medical intervention their illness could not be cured.

"P: It took me nearly two years to get used to it, you know, just worrying to go back [home] - but I couldn’t. (Indigenous female patient, aged 40–49 years)"

For many Indigenous participants, the eventual “acceptance” of dialysis was accompanied by a somewhat bleak sense of resignation described graphically by one Indigenous patient:

"P: Well, a lot of them know they’re stuck here for life until they die, you know. And they know it, and it’s really upsetting to them. (Indigenous female patient, aged 40–49 years)"

"P: I was thinking sad [about] everything – [I] keep looking back to my family. (Indigenous female patient, age 60–69 years)"

In essence, these participants experienced their lives as being simultaneously saved – and devastated – by dialysis.

## Discussion

As a group, Indigenous patients’ dialysis experiences were shaped by several factors including: late referral and unplanned initiation of dialysis; family separation associated with relocation to an urban centre for treatment; barriers to meeting family and community responsibilities; the physical and psychosocial demands of dialysis; and ineffective communication between care providers and patients. As noted, none of these factors were *only* identified by Indigenous patients. Some non-Indigenous patients also experienced the effects of some factors: some non-Indigenous patients had communication problems; some had a rushed and frightening start to dialysis; some had to deal with family separation and so on. However, in contrast to other patient groups, the majority of Indigenous patients experienced *all or most factors* combining in varying levels of intensity throughout the period of their dialysis. As a result, centre-based hemodialysis presents distinct - and particularly complex - challenges for Indigenous patients. Despite such challenges, the majority of Indigenous participants reported their endeavours to maintain the treatment regimen. However, there was also a sense that patients, isolated from family and support networks, became worn down and lost motivation over time. In a setting of serious illness and limited understanding, a range of responses including alienation, isolation, confusion, frustration, anger, denial, resentment and/or mistrust led some patients to act against their own best interests. Often unknowingly, they were also potentially reducing their chances of being deemed suitable for transplantation or for self-care, home-based dialysis modalities.

A key finding of this study concerns the extent and implications of social hardships on the experience of dialysis treatment for many Indigenous patients. Indigenous participants’ narratives revealed how feelings of alienation and isolation substantially increased the challenge of coping with an already demanding treatment. Our findings also support those of similar studies undertaken with Native Canadian ESKD patients, which found that relocation for dialysis disrupts patients’ social support patterns and creates ongoing psychosocial problems for both patients and their families [17,18]. All these more recent findings extend those of an earlier qualitative study exploring the illness and treatment experiences of Aboriginal ESKD patients in a specific region, Central Australia, in which patients similarly reported the stress of reconciling family and cultural responsibilities with treatment requirements [10]. Devitt and McMasters found markedly divergent views between medical staff and Aboriginal patients about the meaning, purpose and priority of adhering to dialysis treatment requirements. Describing the centrality of family and kin relationships in the social and cultural life of Indigenous people (including patients) they say:

*Non-Aboriginal [health] carers rightly exhort patients to focus on their own health requirements. But many are apparently unaware of the enormity of the patient’s own dilemma who seeks to have not only life rather than death, but a life that has meaning in their terms. [*[10]*, p.S114]*

In constructing *“a life that has meaning in their terms”,* Indigenous dialysis patients draw on a cultural and social wellspring that differs greatly from that of their carers and, importantly, includes *specific and different notions of illness causality as well as ideas of how health and well-being are achieved.*

A second key finding of this study was the degree to which miscommunication and/or ineffective communication accounted for patients’ treatment-related difficulties. When combined with relocation and family separation, social dislocation and unplanned treatment initiation, communication difficulties often took patients to the breaking point. Language differences, the failure to use interpreters routinely, and staff - particularly specialists - using inaccessible medical language added further to dissatisfaction and potential disengagement among Indigenous patients. Low levels of literacy and education also hampered effective communication and knowledge transfer. Patients confirmed their difficulties in understanding their situation, treatment and options. Some even perceived this as evidence of staff reluctance to share the “full story” with patients. These findings are in line with our previous research, which found that many Indigenous patients feel uninformed about their illness and eager for more information [19]. There are also parallels in our findings with those of an earlier Australian study investigating effectiveness of communication between Aboriginal ESKD patients and healthcare workers, which found that the pervasive miscommunication between patients and providers often went unrecognised by both groups [20]. Cass and colleagues highlighted the importance of developing shared understandings incorporating perspectives of both medical staff and patients, as well as the importance of understanding clinical communication within a broader social, cultural and political context [20]. As articulated by Humphery and colleagues, the efficacy of clinical communication is constrained by structural issues including poverty, dispossession, marginalisation, limited education, and racial discrimination [21], all of which are relevant to Indigenous Australians.

The findings of this study indicate that dialysis treatment is likely to be more challenging for Indigenous patients than for other patients. Yet our findings also suggest that the large majority of Indigenous participants see themselves as either managing a difficult situation or persevering under very onerous circumstances. This may not add up to the “compliance” proposed by staff but neither could it be said to constitute “non-compliance”. In contrast with views expressed by health professionals [12], we found little evidence of Indigenous patients admitting to intentionally deviating from their dialysis regimen, and any such deviations tended to represent either temporary rebellion or “giving up” after a long period on dialysis. However, it is also possible that Indigenous patients who do not adequately understand their situation, including their treatment requirements, may be unaware how and why their behaviour deviates from expectations. Our findings suggest that most Indigenous patients see themselves as engaging with their dialysis treatment as best they can under fraught circumstances. Nevertheless it also appears that there may be a gap between patients’ views of their own efforts and the assessments of “compliance” by health professionals using a range of clinical measures (such as dialysis attendance records, weight checks and blood tests). This is critical, because, despite recognising that it is both poorly conceptualised and poorly measured, clinicians rely strongly on notions of “compliance” in determining access to transplantation [11,12].

This study, which draws upon self-reported views, attitudes and experiences of 241 Indigenous and non-Indigenous ESKD patients, has some potential limitations. Most importantly, patients’ self-reports can only represent a partial account of the situation. For example, some patients may have over-estimated the extent to which they followed the regimen, particularly if they thought this could affect their chances of being wait-listed for transplantation; others may not have been sufficiently well-informed to assess whether their behaviour accorded with expectations. These limitations aside, the value in garnering the personal experiences and perspectives of a large number of dialysis patients is the rare insight it affords into challenges and obstacles faced by Indigenous patients in maintaining their dialysis treatment.

## Conclusions

The impact that social and situational circumstances have on the ability of Australian Indigenous dialysis patients to fully engage with treatment has important implications for health services. While factors such as place of residence, educational attainment and language are not easily modifiable, treatment practices can be adjusted more readily. Earlier diagnosis could reduce some of the trauma associated with unplanned start to dialysis. Some progress has been made, with late referral declining from 33.8% of Indigenous patients starting treatment in 2005 to 21.2% in 2009 [1], but delayed diagnosis remains an important concern. Improved linkages between specialist renal services and primary care in regional settings would support patients to better prepare, and more effective communication and patient education would significantly strengthen patient understanding of their situation. As well as reviewing the weight placed on patient “compliance” in decision-making, transplant and home dialysis programs should establish more systematic, transparent approaches to “compliance” in their guidelines.

## Competing interests

The authors declare that they have no competing interests.

## Authors’ contributions

JC participated in the overall management and design of the IMPAKT study, in data analysis and interpretation, and in writing and editing the manuscript. KA coordinated data management, conducted interviews, participated in the coding, analysis and interpretation of data, and drafted the manuscript. JD participated in the design of the IMPAKT interview study protocol, and coordinated and participated in data collection, management, coding, analysis and interpretation. CP participated in study design, coordinated the Indigenous community engagement component, and participated in data collection, management, analysis and interpretation. AC conceived of the study, participated in design and overall management and in data analysis and interpretation. All authors participated in the drafting and/or critical revision of the manuscript and read and approved the final version to be published.

## Pre-publication history

The pre-publication history for this paper can be accessed here:

http://www.biomedcentral.com/1471-2369/13/114/prepub
